# A home-based exercise intervention to increase physical activity among people living with HIV: study design of a randomized clinical trial

**DOI:** 10.1186/1471-2458-13-502

**Published:** 2013-05-24

**Authors:** Jason R Jaggers, Wesley Dudgeon, Steven N Blair, Xuemei Sui, Stephanie Burgess, Sara Wilcox, Gregory A Hand

**Affiliations:** 1 Department of Exercise Science, University of South Carolina, Columbia, SC, USA; 2 Department of Health, Exercise and Sport Sciences, The Citadel, Charleston, SC, USA; 3 School of Nursing, University of South Carolina, Columbia, SC, USA; 4 Department of Epidemiology and Biostatistics, University of South Carolina, Columbia, SC, USA; 5 Arnold School of Public Health, PHRC, 921 Assembly St, Columbia, SC, 29208, USA

**Keywords:** Randomized trial, Cardiovascular disease, HIV, Physical activity, Self care, Study design

## Abstract

**Background:**

While combination antiretroviral therapy has extended the life expectancy of those infected with human immunodeficiency virus (HIV), there is a high prevalence of comorbidities that increase the risk of cardiovascular morbidity and mortality among people living with HIV/AIDS (PLWHA). The side effects associated with antiretroviral therapy (ART) lead to multiple metabolic disorders, making the management of these metabolic issues and risk of cardiovascular disease (CVD) in those treated with ART a critical issue. Clinical research trials, primarily clinical exercise, rarely include this population due to unique challenges in research methods with underserved minority populations living with a life threatening illness like HIV/AIDS. This paper describes the rationale and design of a randomized clinical trial evaluating the feasibility of a home-based exercise program designed to increase physical activity (PA) and reduce the risk of CVD in PLWHA.

**Methods/design:**

PLWHA being treated with ART will be randomly assigned to one of two groups: a home-based PA intervention or standard care. All participants will receive an educational weight loss workbook and pedometer for self-monitoring of PA. Only those in the intervention group will receive additional elastic Thera-bands® for strength training and behavioral telephone based coaching.

**Discussion:**

This study will evaluate the feasibility of a home-based program designed to increase PA among PLWHA. Further, it will evaluate the effectiveness of such a program to decrease modifiable risk factors for CVD as a secondary outcome. This study was funded by the NIH/NINR R21 Grant 1R21NRO11281.

**Trial registration:**

Clinical Trial Identifier NCT01377064

## Background

People living with HIV/AIDS (PLWHA) are at an increased risk for cardiovascular disease (CVD) once they begin taking antiretroviral therapy (ART). Studies that control for traditional CVD risk factors show a detrimental effect of ART on CVD risk in PLWHA. For example, the incidence of myocardial infarction (MI) increases directly with increased exposure to specific ART regimens [1,2]. The majority of known side effects associated with ART directly alter metabolic processes, thus increasing the risk of metabolic syndrome. Symptoms of these side effects include lipodystrophy (abnormal fat displacement), hyperlipidemia (i.e. triglycerides, cholesterol), decreased HDL-C, and impaired fasting glucose among others [3,4]. Similar symptoms are commonly observed among people known to be at an increased risk for CVD, diabetes, and other chronic diseases.

Extensive research has established the benefits of routine PA in the general population, including decreased risk of CVD [5]. PA is an appropriate intervention for reducing many modifiable risk factors of CVD, including a number of those that are elevated with HIV infection and/or treatment. Studies have shown that people of all ages infected with HIV have abnormally low levels of cardiorespiratory fitness (CRF) [6]. These reductions have been attributed to sedentary behavior and lifestyle habits. Further, PLWHA often exhibit a maximal VO2 of 24% - 44% below their age-predicted normal values [6]. CRF, as determined by a maximal VO2 test, is a powerful predictor of all-cause and CVD mortality[7,8] and of type 2 diabetes mellitus [9]. Short duration studies have shown significant increases in CRF with moderate or high intensity PA training in as little as 6 to 12 weeks as well as a dose response to aerobic activity [6,10]. Other beneficial physiological and anthropomorphic adaptations include increased serum HDL-C, decreased triglycerides and total cholesterol [11,12], and reduced BMI, waist-to-hip ratio, and overall body fat [13]. Recent studies show that muscular strength is inversely associated with risk for metabolic syndrome and all-cause mortality [14,15].

PA interventions that require regular attendance at a facility are not ideal for populations that are more likely to be financially disadvantaged, such as PLWHA, due to barriers related to transportation, access, and cost. We have used home-based PA programs successfully with various populations [16-18]. These programs have targeted healthy populations and those with heart failure, cancer, sporadic inclusion body myositis (IBM), chronic obstructive pulmonary disorder (COPD), and outcomes associated with various inflammatory responses [19-22]. Other trials have focused on functional outcomes such as the ability to complete tasks of daily living, or overall cardiorespiratory fitness [23,24]. These programs incorporated moderate intensity PA, which has been proven to be safe for clinical populations with functional limitations [23,24]. Telephone delivered home-based interventions have been found to be effective in promoting PA in general populations of adults, and this intervention approach has been recommended for wider dissemination [25]*.* The purpose of this paper is to describe the detailed methods for a randomized clinical trial of a telephone delivered home-based PA program designed to increase physical activity and reduce the risk of CVD in PLWHA.

## Methods

This randomized clinical trial is being conducted to test the effectiveness a 9-month home based PA intervention. Further, we will examine the effects of routine PA relative to a standard of care group. Physical and psychological testing, as well as blood sampling, is being performed on each participant at baseline, 18 weeks, and 36 weeks. Measurements will include either direct measurement or measurement of surrogate markers/indices for body composition, stress and health-related quality of life, blood lipids and inflammatory markers, and glucose metabolism.

### Description and selection criteria for participants

All participants were recruited between 2010–2012 from one of two sites via physician reference or word of mouth; the greater Columbia, South Carolina area or the Charleston, South Carolina area. Study staff recruited underactive men and women 18 years of age or older with a positive HIV serostatus and on ART. Inclusion and exclusion criteria for this study are shown in Table 1.

**Table 1:** Inclusion/exclusion criteria

**Inclusion criteria**	
• Age 18 years and older	
• Medical diagnosis of HIV-1 positive serostatus	
• Sedentary lifestyle: not actively exercising ≥ 3 d · wk^-1^ for 20 min per session	
• Stable, DHHS-approved ART regimen for previous 3 months, with HIV viral load below 75 copies/mL	
• Capable of performing the required exercise regimen	
• Have daily access to a telephone for approximately 10 months	
• Capacity and willingness to provide informed consent and accept randomized group assignment	
**Exclusion criteria**	**Temporary exclusion criteria**
• Individuals who have a clinical history strongly suggestive of Type 1 diabetes.	• Total cholesterol ≥240 mg/dl with LDL-C ≥160 mg/dl or TG levels ≥300 mg/dl. Note: Individuals on cholesterol lowering medications but meeting blood lipid requirements are eligible.
• History of serious arrythmias, cardiomyopathy, congestive heart failure, stroke or transient ischemic cerebral attacks, peripheral vascular disease with intermittent claudication, myocardial infarction, or CABG.	• Resting blood pressure >160 systolic and/or 90 diastolic. Note: Individuals on blood pressure medications but meeting blood pressure criteria are eligible.
• Malignancies in the past 5 years, except therapeutically controlled skin cancer.	• HbA1c ≥11%. Individuals whose HbA1c exceeds this level will be recommended to seek treatment immediately. Such individuals may be re-screened after three months to re-assess HbA1c eligibility.
• Plans to be away > 4 weeks in the next 9 months.	• Current opportunistic infection at the time of screening.
• Score of 5 or greater on the DAST or MAST (signifying excessive use of drugs or alcohol).	• Participation in another intervention trial.
• Weight loss in excess of 10% body weight in previous 12 weeks.	• Other temporary intervening event, such as sick spouse, bereavement, or recent move.
• Chronic or recurrent respiratory, gastrointestinal, neuromuscular, neurological, or psychiatric conditions.	
• Inflammatory-related conditions such as collagen disorders.	
• Any other medical condition or disease that is life-threatening or that can interfere with or be aggravated by exercise.	

### Participant screening

**Figure 1:**
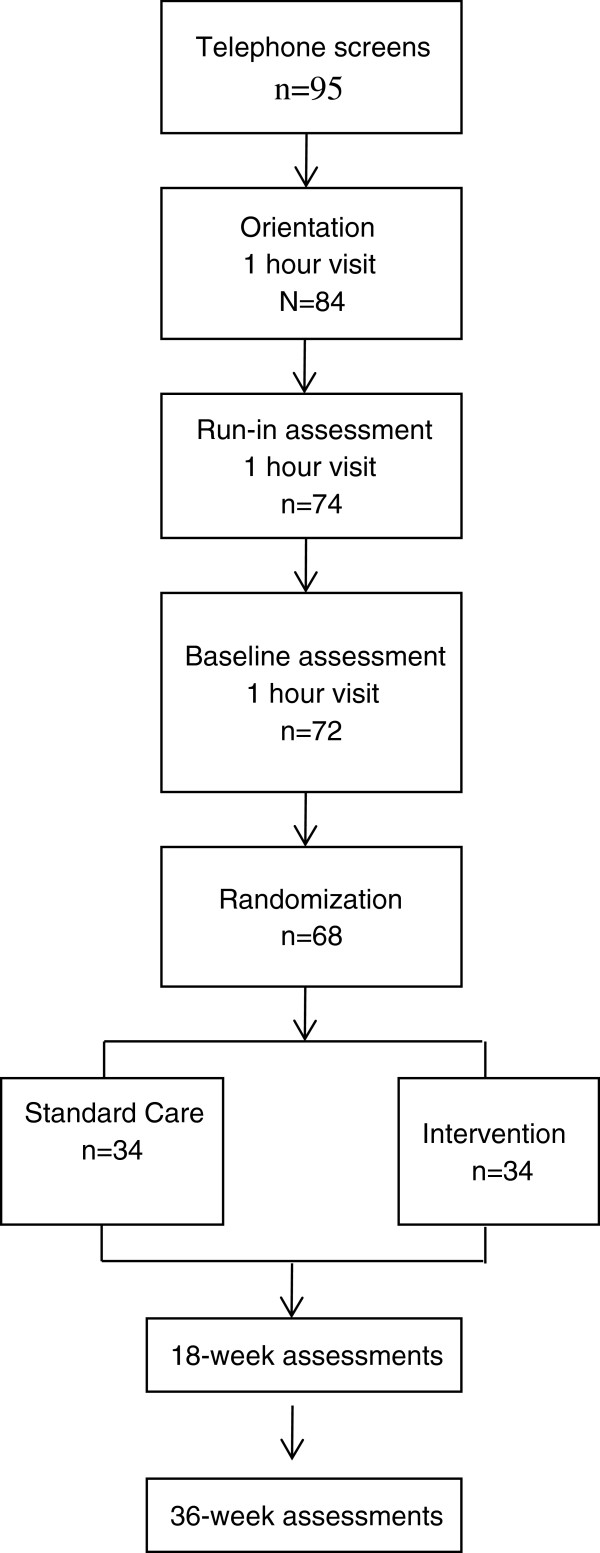
Study flow chart.

Figure 1 shows a flow chart of the study as participant’s progress through, beginning with an initial telephone pre-screening interview. Potential participants were asked general eligibility questions regarding their age, HIV status, current ART prescription, and telephone access over the next 10 months. All eligible participants who wished to learn more about the study were invited to a study orientation session.

At the study orientation, a staff member gave a slide presentation explaining the purpose of the study and study expectations of participants. This presentation included the participant responsibilities and potential health benefits and risks related to study participation. Participants were given an opportunity to ask questions about the study and those who wished to participate signed an informed consent approved by the University of South Carolina’s Internal Review Board and completed further questionnaires requesting contact and demographic information. All eligible participants who signed the informed consent were scheduled to take part in a run-in period.

During the run-in period participants were asked to complete additional self-report questionnaires as described below. Trained staff also obtained the following standardized measurements: resting blood pressure, height, weight, waist and hip circumference, and a fasted blood draw. To avoid measurement error, each measurement is obtained in triplicates with the average of the two closest as the primary value used for data collection. All participants are instructed not to eat or drink anything except water at least 8 hours prior to their scheduled run-in visit. At the end of the session participants were given a SenseWear® armband designed to measure daily PA with oral and written instructions for proper wear. Participants were instructed to wear the armband at all times except while bathing or swimming until they returned for baseline testing. The run-in activities serve several purposes. First, wearing an armband allows participants to better understand what type of activities would be required in the study and to see if the participant would be compliant with attending multiple study visits, as required by the trial. The goal is to screen out participants unlikely to adhere to study requirements. The second reason for including the run-in period and session is to reduce participant burden at the orientation and baseline assessments by spreading the questionnaires across multiple visits.

Participants returned for their baseline session 7–10 days after the run-in. At this time the armband was collected and PA data was immediately uploaded into the dataset. Participants are not provided with any data retrieved from the armband at this time. During this session all participants completed a maximal exercise stress test with a 12-lead ECG and breath by breath oxygen analyzer. All tests were completed on a treadmill and supervised by the study medical director. Following successful completion of the exercise test and clearance for participation by the medical director, study participants were randomized.

### Randomization

After successfully completing all screening visits (orientation, run-in, and baseline assessment), eligible participants were randomized into 1 of 2 groups: standard care or intervention. Both groups received an evidence-based book on healthful eating and regular physical activity [26] and a pedometer. Those randomized into the intervention group received a one-on-one PA training session to explain in further detail participant expectations and demonstrate proper use of the elastic Thera-Bands® for strength training. The physical activity education session provides an overview of what constitutes physical activity, benefits of physical activity, different types of physical activity, current physical activity recommendations, tips for starting a physical activity program and proper use of the physical activity log. In addition to the education session the health educator worked with each participant to help them establish reasonable short term goals that would eventually lead up to a common long term goal among the participants (≥ 150 minutes of routine PA per week and 2–3 resistance training sessions per week).

### Follow-up examinations

Participants are scheduled to return for a follow-up data collection visit that occurs at 18- and 36- weeks after randomization. The same measurements and questionnaires completed during the run-in session are assessed during each follow-up visit. All sessions are completed in the morning following an 8 hour fast. Participants are also given the armband to wear for 7–10 days, with detailed oral and written instructions for proper use. Participants are scheduled to return 7–10 days later to return the armband.

#### Outcome measures and methods

The primary outcome of interest is physical activity, as measured by the SenseWear® armband. These measurements will be used as an index of program adherence as well as a measure of total physical activity. Secondary outcomes include physiological and psychological variables, such as cardiorespiratory fitness, adiposity and fat distribution, full blood lipid panel, depression, anxiety, stress, and health-related quality of life. Tertiary exploratory outcomes include assessment of serum CRP and IL-6.

### Physical activity assessment

Physical activity will be assessed with the SenseWear® armband, a commercially available (http://www.bodymedia.com) lightweight physical activity monitor that is worn on the upper left arm halfway between the acromion and olecranon processes. Using tri-accelerometry technology augmented by 2 heat sensors (a thermistor-based skin surface sensor and a proprietary heat flux sensor), and a galvanic skin response sensor, the device is able to accurately measure and record total energy expenditure, and also measures activity intensity. These 4 internal sensors turn on the monitor when detecting skin contact and measures total time the armband was worn, daily energy expenditure, step count, sleep efficiency, and the intensity, duration, and frequency of physical activity bouts. All armband data will be analyzed by computer-based software using demographic information (gender, age, height, and weight at prior assessment) and proprietary algorithms.

### Validity of SenseWear armband

Validity of armband energy expenditure estimates has been reported in several conditions including resting, exercise (ie treadmill and cycle ergometry), and free-living conditions (ie physical activity and exercise) [27-29]. St-Onge et al. found that when comparing the armband to doubly labeled water daily energy expenditure had an interclass correlation of 0.81 (P < 0.01) between the two methods [30]. When comparing the armband to the Intelligent Device for Estimating Energy Expenditure and Activity (IDEEA) Welk et al. showed comparable results between the armband and IDEEA when estimating energy expenditure and PA [29].

### Intervention

#### Components and approach

Two interventionists were trained by a clinical psychologist with expertise in behavioral interventions to implement the Active Choices program (developed at Stanford University). Active Choices is a home-based physical activity program that uses theoretically-based strategies to help participants make lifestyle changes. Active Choices is based on both social cognitive theory [31] and the transtheoretical model [32]. The intervention begins with a 60-minute individual face-to-face session in which rapport was established; the program and expectations are described; a PA and diet history is taken; realistic, specific, and measurable short-term and long-term goals are set; safety information is provided; instructions for wearing a pedometer is given; self-monitoring tools (pedometer and log) are distributed; and a schedule for follow-up telephone calls is set.

The face-to-face session will be followed by regular telephone counseling calls (15–20 minutes each). During the initial adoption phase (weeks 1–8) participants will receive telephone calls once a week. After the adoption phase, participants will enter a transition phase (weeks 9–26) during which participants received a telephone call every other week. The goal for the transition phase is for the participants to work up to 150 minutes of moderate- intensity physical activity and complete two resistance training sessions. Following the transition phase, participants begin a maintenance phase (weeks 27 to the end of the trial) where they will continue to receive a telephone call every other week. The exercise regimens of weekly 150 minutes of moderate-intensity PA and 2 resistance training sessions will also continue. Although the *Active Choices* program recommends two calls per month during the first two months and one call per month thereafter, we believe that more regular contact is important with PLWHA. We anticipate that participants in our trial will be predominantly financially disadvantaged, have many challenges to changing behavior, and would benefit from additional support. More frequent contact also allows us to monitor participant health and safety.

Each call will begin with a review of goals (cognitive or behavioral) set on the last call, assessment of current health and medical status and changes in status, and actual physical activity participation since the last call (type of activities and total time spent doing each activity). Participants will be encouraged to discuss barriers to change (and the interventionist helped to engage the participant in problem solving skills) as well as successes. The participant’s stage of readiness for change will be assessed and this information, along with information learned about barriers and successes on previous calls help to guide the selection of counseling topics/strategies for the call. For example, discussion of a cognitive strategy such as becoming more aware of the benefits of PA could be used for participants in precontemplation or contemplation, whereas a behavioral strategy such as enlisting social support could be discussed for participants in later stages of change (preparation, action, maintenance). The interventionist will be provided a list of call topics (i.e., cognitive or behavioral strategies and approaches that map onto the two theories) most appropriate for each stage of change. Over the course of the intervention, the interventionist will be encouraged to choose and discuss multiple call strategies that are most relevant for the participant. Calls typically end with the interventionist helping the participant select cognitive or behavioral goals for the next one to two weeks and discussing ways to overcome barriers that may get in the way of these goals. The interventionist may send a “tip sheet” if they feel that participant needs additional information (e.g., exercising during the heat).

Frequent self-monitoring of physical activity, diet, and weight will be strongly encouraged, as self-monitoring is positively associated with behavioral adherence (32). A pedometer will be used as a motivation and self-monitoring tool. The health counselor will use minutes per day of physical activity as part of goal setting activities (e.g., focusing on increasing weekly physical activity time through 10-minute or more bouts of walking).

##### Non-exercise standard care group

Since randomization occurred after the run-in period, the participants in the standard care group received the same educational material and weight loss manual just as those randomized to the PA group. Physical activity habits will be monitored throughout the study for participants randomized to the standard care group in the same manner as for the intervention group using the SenseWear® armband. One potential problem in this group is the possibility of participants losing interest in the study because they are assigned to a “control” group. We have been successful in reducing standard care group dropout in previous studies by maintaining telephone contact with standard care group participants. In this study, we will contact the standard care group participants by telephone every two weeks to update health status, maintain current contact information, and emphasize the importance of their participation for a successful study.

### Testing procedures

#### Graded exercise stress test

Following a medical examination including evaluation of family and personal medical history, resting blood pressure, and resting ECG evaluation, each participant completed a graded exercise stress treadmill test (GXT). The GXT is used to screen for abnormal physiological responses to exercise, including blood pressure and ECG responses, and for determining the participant’s current level of cardiorespiratory fitness. The test is terminated either at volitional exhaustion or at observation of abnormal responses that are contraindications for exercise. This test has been found to be highly reproducible with a high sensitivity to changes in VO2 peak [33].

#### Body mass index and visceral fat

Anthropomorphic measurements are made using standard laboratory equipment. Height and weight will be used to determine body mass index (BMI, weight in kg/height in meters^2^) as an index of total body fat. Waist circumference (WC) is measured as an index of visceral fat. Waist circumference is only an indirect measure of visceral fat, but budget constraints preclude more direct measurement of visceral fat. Nevertheless, combined use of BMI and waist circumference has been shown to be a good predictor of mortality [34].

### Laboratory assays from blood products

#### Blood sampling

Blood draws are performed following an 8 hour fast. Participants will be asked to refrain from taking aspirin and other anti-inflammatory medications for 48 hours prior to the blood draw. Twenty milliliters of whole venous blood will be drawn into standard vacutainer tubes by trained personnel. Standard separation procedures will be used for collection of serum and plasma. Samples will be stored at −80°C until future analysis.

#### Blood analysis

The blood components of interest will be measured in duplicate by LabCorp® clinical trials division. All samples are being analyzed by a LabCorp professional at a central location that undergoes rigorous quality assurance testing to deliver high accuracy and reliability in the results.

### Self-report questionnaires

#### Drug abuse screening test (DAST)

The DAST is a 28-item self-report questionnaire that provides quantitative index of problems related to drug misuse [35]. The DAST has been validated with an internal consistency of .92 [35]. A score of 6 or greater is suggestive of significant drug misuse which was used as part of the exclusion criteria.

#### Michigan alcohol screening test (MAST)

The MAST is a 25-item self-report questionnaire that provides rapid and effective screening for alcohol-related problems and alcoholism. Test-retest reliability for the MAST has been reported as .84 [36], however, Boyd reported a reliability of .94 in rural women substance abusers [36]. A score of 5 or greater is suggestive of significant alcohol dependency which was used as part of the exclusion criteria.

#### Sociodemographic data

Sociodemographic characteristics was obtained to provide a description of the participants in the study and to assess the generalizability of the results. Variables include age, gender, race, income, educational level, employment and marital status, as well as the number of children living in the household and previous medical conditions. Additionally, information was collected from the participants to describe the usual care (pharmacological and psychological) they receive to control this as a potential confounder.

#### Medication

Because medication use and change in medications could be a confounding factor, all medications will be recorded by self-report, including drug name and specific dosage, at the 0, 18, and 36 week time points. Medication changes that are determined to be significant will be included as covariates during data analysis, however changes in medications will not exclude or drop the participants from the study.

#### Perceived stress scale (PSS)

The 10-item PSS assesses the degree to which an individual finds life events unpredictable, uncontrollable, or overwhelming [37]. Internal consistency has been demonstrated by a Cronbach’s alpha of 0.78 [37]. Validity of the PSS is supported by significant correlations with greater help-seeking, poorer health, more health service utilization, and poorer life satisfaction [37].

#### MOS 36-item short-form health survey (SF-36)

The SF-36 is a widely-used and validated self-report measure of health-related quality of life [38,39]. It consists of eight health concepts including physical function, social function, pain, mental health, energy/fatigue, general health perceptions, role limitations due to physical problems, and role limitations due to emotional problems. The SF-36 can be divided into the two parts: the Mental Composite Score (MCS) and the Physical Composite Score (PCS). The internal consistency reliability coefficients are high for its eight scales and ranges from 0.78 to 0.93 [40].

#### Centers for epidemiologic studies-depression (CES-D)

The 20-item CES-D is a widely-used and validated self-report measure of depressive symptoms [41,42]. Components of the CES-D include depressed mood, feelings of guilt and worthlessness, feelings of hopelessness and helplessness, psychomotor retardation, loss of appetite, and sleep disturbance. The CES-D has a high internal consistency in both the general population (.85) and in patient populations (.90) [41].

### Theory-based measures

Several self-report measures were chosen to measure the theoretical constructs which form the basis of our intervention. These measures will allow us to examine whether the intervention changed constructs that are proposed by social cognitive theory and the transtheoretical model to be mediators of change. While it would be ideal to collect data on all of the constructs from these two theories, we have selected to focus on those most widely used in intervention studies in order to reduce participant burden.

The Self-Efficacy for Exercise Questionnaire consists of 14 items that ask participants to rate their confidence to be physically active (ranging from 0% to 100%) when faced with commonly-cited barriers (e.g., when tired, depressed, have a lot of work to do, etc.) [43]. This questionnaire has been shown to be valid and reliable across race, education, gender, and weight categories [44]. Internal consistency was high for all subgroups (α = .90 to .94) and criterion-related validity was established across subgroups.

Social support for PA will be assessed with a commonly-used 13-item scale developed by Sallis and colleagues [45]. This scale has show acceptable test-retest reliability (r = 0.55 to 0.86) and internal consistency (α = 0.61 to 0.91), as well as concurrent criterion-related validity [45].

Self-regulatory behavior will be assessed with a 20-item scale that assesses the degree to which participants self-monitor, set goals, and problem solve [46]. The measure has been shown to have high internal consistency (α = .89 for goals, .87 for plans) and strong associations with PA [46].

Decisional balance will be assessed with a commonly used 16-item (10 pros, 6 cons) scale developed by Marcus and colleagues [47]. This scale has shown acceptable internal consistency (α = 0.79 for cons; 0.95 for cons) and significant associations with stage of readiness for change.

Finally, the five cognitive and 5 behavioral processes of change will be measured with a commonly-used 20-item scale (2 items for each process) developed by Marcus and colleagues [48]. Factor analysis has revealed two higher order constructs (cognitive/experiential and behavioral processes), and those in early stages of change report greater use of cognitive processes and less use of behavioral processes, and vice versa for those in later stages [48].

### Statistical analysis

Descriptive baseline characteristics for group comparisons will be tabulated as means and standard deviations (SD) or as percentages. Differences between the two groups in the primary outcome will be tested according to the intention-to-treat philosophy. All randomized participants will be analyzed according to their group assignment at randomization, regardless of adherence to the intervention. The primary comparison is the difference in the amount of moderate-intensity physical activity between the intervention group and the standard care group at 18 and 36 weeks of the intervention, respectively. Secondary, tertiary, and exploratory analyses will focus on the other outcomes and subgroups, and findings will be interpreted cautiously. All analyses will take into account pre-specified covariates, including age, gender, race, education, smoking status, response sets, medication changes, and baseline values of outcome measures. Analyses of continuous outcome measures will be based on analysis of covariance (ANCOVA) models of 18- and 36-month change scores since baseline and treatment effects will be summarized as least-squares adjusted means [49]. Analysis of binary outcomes will be based on logistic regression, and analyses of ordered polytomous outcomes will be based on ordered logit proportional-odds models [50]. The potential effects of missing data will be explored under various models for nonignorable missing data mechanisms, and through multiple imputation models under ignorable missing data assumptions [51]. An α level of 0.05 will be used to indicate statistical significance.

### Power calculation

For a feasibility study, power is not a major consideration. However, being able to detect moderately large differences between groups is reasonable. Assuming a Type I error rate of α = 0.10 and 80% power, we can detect a difference between the intervention and control groups in the change in PA of 0.55 standard deviation units. That is, if the standard deviation of accelerometry-based PA is 120 min/week MPA and the pre-post correlation is 0.4, we can detect a difference of 80 minutes/week. A major goal of this study is to generate data to inform development of a larger randomized trial of this community-based PA intervention. The data will therefore be used to generate descriptive statistics (means and standard deviations) for the various physiological risk factors for cardiovascular disease and their corresponding activity-induced changes across the intervention in this unique population.

### Baseline results

#### Participant recruitment

Figure 1 shows the flow of participants through the recruitment and randomization phase of the study. A total of 95 phone screenings were conducted, of which 84 were considered eligible after preliminary screening and invited to attend the orientation session. The most common reason for ineligibility following the phone screen included not currently on ART. Of those invited to attend the orientation session, 8 were no shows and could not be re-scheduled, 2 were found ineligible, and 74 were scheduled to return for the run-in visit for further data collection. The total number of participants who attended the run-in session was 72, with 2 no shows, in which all who attended were invited to the final baseline session. Prior to randomization, but after the baseline measurement was conducted, 3 participants (4%) were further excluded due to uncontrollable hypertension which is a contraindication to exercise testing. Any participant unable to complete the graded exercise test was not cleared for study participation by the medical supervisor. Thus, 94% of those consented and measured at baseline were randomized to an intervention condition.

#### Participant characteristics

Demographic and health-related baseline characteristics of the study sample are presented in Table 2. Intervention and Control participants did not differ significantly (p > 0.05) on any characteristic at baseline. Most of the participants self-identified as African-American (82.4%) and female (54.4%). The average age was 40 years and most participants were either singe/never married (39.7%) or divorced (17.6%). Although majority have some college education (41.2%), only 19% of the study participants have a college degree and more than half are out of work by self-identifying as either unemployed (28%) or unable to work (25%).

**Table 2:** Baseline characteristics of participants

**Characteristic**	**Intervention (n = 34)**	**Control (n = 34)**	**Total (n = 68)**
Gender, % (n)			
Male	47.1 (16)	44.1 (15)	45.6 (31)
Female	52.9 (18)	55.9 (19)	54.4 (37)
Race, % (n)			
White	14.7 (5)	8.8 (3)	11.8 (8)
Black/African American	79.4 (27)	85.3 (29)	82.4 (56)
>1 category	2.9 (1)	2.9 (1)	2.9 (2)
Hispanic ethnicity, % (n)			
Yes	2.9 (1)	5.9 (2)	4.4 (3)
No	97.1 (33)	94.1 (32)	95.6 (65)
Age in years, Mean ± SD	49 ± 11	48 ± 10	48 ± 10
Education, % (n)			
Never attended school	0	0	0
Grades 1-8	0	0	0
Grade 9-11	14.7 (5)	14.7 (5)	14.7 (10)
Grade 12 or GED	23.5 (8)	26.5 (9)	25.0 (17)
Some college	38.2 (13)	44.1 (15)	41.2 (28)
College grad	23.5 (8)	14.7 (5)	19.1 (13)
Maritial status, % (n)			
Married	8.8 (3)	11.8 (4)	10.3 (7)
Divorced	23.5 (8)	11.8 (4)	17.6 (12)
Widowed	5.9 (2)	8.8 (3)	7.4 (5)
Separated	11.8 (4)	11.8 (4)	11.8 (8)
Never married	47.1 (16)	32.4 (11)	39.7 (27)
Couple/Significant Other	2.9 (1)	23.5 (8)	13.2 (9)
Employment status, % (n)			
Employed	29.4 (10)	44.1 (15)	36.7 (25)
Not employed	35.3 (12)	20.6 (7)	28.0 (19)
Student	2.9 (1)	5.9 (2)	4.4 (3)
Retired	2.9 (1)	2.9 (1)	2.9 (2)
Unable to work	29.4 (10)	20.6 (7)	25.0 (17)
Income, % (n)			
$0-9,999	29.4 (10	35.3 (12)	32.4 (22)
$10,000- 19,999	26.5 (9)	20.6 (7)	23.5 (16)
$20,000- 29,999	14.7 (5)	11.8 (4)	13.2 (9)
$30,000- 39,999	5.9 (2)	17.6 (6)	11.8 (8)
$40,000- 49,999	2.9 (1)	2.9 (1)	2.9 (2)
$50,000- 59,999	0	2.9 (1)	1.5 (1)
Prefer not to say	20.6 (7)	8.8 (3)	14.7 (10)
Children in household, % (n)			
0	85.3 (29)	76.5 (26)	80.9 (55)
1	2.9 (1)	11.8 (4)	7.4 (5)
2	5.9 (2)	5.9 (2)	5.9 (4)
3	0	2.9 (1)	1.5 (1)
≥ 4	5.9 (2)	0	3 (2)
Weight in pounds, Mean ± SD	191.48 ± 58.59	192.14 ± 49.38	191.81 ± 53.76
BMI (kg/m^2^), Mean ± SD	30.04 ± 8.38	30.79 ± 7.90	30.42 ± 8.09
Waist circumference, Mean ± SD	98.74 ± 20.96	99.22 ± 18.54	98.98 ± 19.63
Blood Pressure, Mean ± SD			
Systolic (mmHg)	127 ± 16	130 ± 14	128 ± 15
Diastolic (mmHg)	83 ± 10	83 ± 10	83 ± 10
Self-reported conditions %(n)			
Heart condition			
Yes	0	0	0
No	100 (34)	100 (34)	100 (68)
Cancer			
Yes	2.9 (1)	8.8 (3)	6 (4)
No	97.1 (33)	91.2 (31)	94 (64)
Diabetes			
Yes	5.8 (2)	20.6 (7)	13.2 (9)
No	94.2 (32)	79.4 (27)	86.8 (59)
High Cholesterol			
Yes	36.4 (12)	33.3 (11)	34.8 (23)
No	63.6 (21)	66.7 (22)	65.2 (43)
Hypertension			
Yes	45.5 (15)	48.5 (16)	47 (31)
No	54.5 (18)	51.5 (17)	53 (35)
Sleep apnea			
Yes	17.6 (6)	6.1 (2)	11.9 (8)
No	82.4 (28)	93.9 (31)	88.1 (59)
Arthritis			
Yes	20.6 (7)	12.1 (4)	16.4 (11)
No	79.4 (27)	87.9 (29)	83.6 (56)
Hepatitis C			
Yes	17.6 (6)	15.6 (5)	16.7 (11)
No	82.4 (28)	84.4 (27)	83.3 (55)
Fasting blood analysis, Mean ± SD			
Cholsterol (mg/dL)	188.89 ± 52.92	186.59 ± 38.55	187.76 ± 46.01
Triglycerides (mg/dL)	156.39 ± 146.87	130.59 ± 84.44	143.73 ± 119.95
LDL (mg/dL)	109.56 ± 35.98	107.41 ± 36.68	108.48 ± 36.00
HDL (mg/dL)	45.86 ± 20.58	52.96 ± 24.60	49.35 ± 22.71
VLDL (mg/dL)	26.19 ± 11.14	26.22 ± 16.90	26.20 ± 14.17
Glucose (mg/dL)	106.93 ± 48.22	111.33 ± 34.41	109.09 ± 41.69
C-Reactive Protein (mg/L)	4.76 ± 5.53	5.49 ± 5.96	5.12 ± 5.70
Interleukin-6 (pg/mL)	5.16 ± 7.11	4.07 ± 1.95	4.55 ± 4.88

## Discussion

While combination antiretroviral therapy has dramatically extended the life expectancy of those infected with human immunodeficiency virus (HIV), there is a high prevalence of comorbidities that increase the risk of cardiovascular morbidity and mortality in the general population, and people living with HIV/AIDS (PLWHA) [52,53]. The side effects associated with antiretroviral therapy (ART) lead to multiple metabolic disorders, making the management of these metabolic issues and risk of cardiovascular disease (CVD) in those treated with ART a critical issue. Studies indicate that even relatively modest increases in physical activity (PA) can increase cardiorespiratory fitness, reduce visceral fat and increase muscle mass, reduce serum total and LDL/VLDL cholesterol and triglycerides, and increase serum HDL cholesterol within the general population [54-56], and similar results have begun to emerge in regards to PLWHA [57,58]. Due to the low socio-economic status of this particular population it is extremely important that a feasible and cost-effective program be implemented, such as routine physical activity, to help them manage the metabolic complications associated with ART. This study will evaluate a telephone delivered home-based PA program designed to increase PA among PLWHA. Further, it will evaluate the effectiveness of such a program to decrease modifiable risk factors for CVD as a secondary outcome. To our knowledge there are no data available on the outcomes, in terms of either program adherence or risk factor reduction, of a home-based PA/exercise training program when prescribed to PLWHA. This gap in knowledge regarding home-based training for community-dwelling patients is of special concern considering the disproportionate prevalence of HIV infection in poor, rural, and minority populations who have limited access to exercise training facilities and professional support.

PA has been shown in center-based clinical trials to reduce CVD risk factors in PLWHA and home-based PA programs have produced benefits in numerous clinical and healthy populations. This study will allow us to test methods and collect process evaluation data with the goal of disseminating an effective approach to increasing PA levels to reduce CVD risk in PLWHA. A secondary goal of this study is to provide a preliminary assessment of activity-induced changes to CVD risk factors including cardiorespiratory fitness, blood lipids and markers of systemic inflammation. Currently this study is in the final stages of data collection. All participants have been recruited and randomized.

## Abbreviations

PLWHA: People living with HIV/AIDS; ART: Antiretroviral therapy; CVD: Cardiovascular disease; PA: Physical activity; BMI: Body mass index; WC: Waist circumference; GXT: Graded exercise test.

## Competing interests

The authors declare that they have no competing interest.

## Authors’ contributions

GAH, SNB, JRJ, WD, and SW conceived of the study, participated in the design and coordination. SB served as on-site medical director and oversaw all aspects of baseline data collection. XS managed the dataset and helped prepare the final dataset for analysis. All authors helped draft the current manuscript. All authors read and approved the final manuscript.

## Pre-publication history

The pre-publication history for this paper can be accessed here:

http://www.biomedcentral.com/1471-2458/13/502/prepub
